# Osteopathy vs. the Medical Age

**Published:** 1898-12

**Authors:** William M. Warren

**Affiliations:** Detroit, Mich.


### Correspondence.

### OSTEOPATHY vs. THE MEDICAL AGE.

DETROIT, Mich., October 31, 1898.

*To the Editor Buffalo Medical Journal:*

*Sir*—I hand you herewith copy of a communication which appears over my signature on pages 626 and 627 of the *Medical Age* for October 25th. I earnestly hope that in my determination to oppose uncompromisingly, in the courts and through every legitimate means at my command, a powerful faction of irregular practitioners, I may count on the approval and coöperation of your valued journal. Very sincerely yours,

WILLIAM M. WARREN.

The following is the communication referred to in the foregoing letter:

### THE LIBEL SUIT OF WILLIAM SMITH, OSTEOPATHIST—A COMMUNICATION FROM THE PUBLISHER.

*To the readers of the Medical Age:*

Dr. William Smith, osteopathist, has a grievance against *The Medical Age*, and demands $25,000 damages.

The ground of his plaint is an editorial reflecting discredit on Dr. Smith, on the *Journal of Osteopathy* and on osteopaths in general. The subject is set forth editorially in *The Medical Age* of September 26, 1898, and a reprint of this editorial will be sent on application.

I need hardly assure any one familiar with the past record of the *Age*, that William Smith, M. D., D. O., has a large contract on his hands. His quest for damages is likely to prove futile, and his armor will need patching if it is to withstand the hard legal knocks that will be showered and battered upon it before he touches one dollar of the *Age's* money!

Pray do not fancy, however, that William Smith and osteopathy are to be lightly dismissed with the contempt that they merit. There is no use in blinking the fact that the lack of efficient organisation amongst reputable medical men has permitted the whole brood of quacks and charlatans to flourish apace. By the strangest irony of fate, osteopathy, in some respects the most grotesque of medical aberrations, has well illustrated Lecky's dictum that a small but cohesive and determined minority can exert a political influence wholly disproportioned to its real weight and numbers.

In Kentucky, thanks to the resolute leadership of a handful of physicians, ably guided by Dr. Mathews, the osteopaths have been driven from the state. Not so, however, in Missouri or—I blush to say it—in Michigan, Vermont, North Dakota, South Dakota, Illinois, Colorado and North Carolina (*American Medico-Surgical Bulletin*). In these more lax and indulgent communities osteopathy boasts its numerous followers, its "schools of instruction," its periodicals of propaganda, its political influence in legislation, its shameful immunity from the penalties by which society properly seeks to rid itself of quackish parasites.

Emboldened by its success, osteopathy now enters the courts and offers battle to a medical journal which disputes its respectability. The challenge is accepted. In the interest of science, in defense of ethical and honorable medicine, in defiance of a quackery that constitutes a deep disgrace to an enlightened age and a stain on the communities which give it shelter, the *Age* proposes to maintain its position and to continue its denunciations of the ignorant pretenders who fatten on the sufferings of the credulous and confiding.

Having put my hand to the plow in this uncompromising fight with quackery, I beg leave to assure you that there will be no turning back.

I need not point out the bearings this contest must have on the interests of legitimate medicine, and I earnestly hope that the *Age* may count on the moral support and commendation of the entire profession.

Faithfully yours,

WILLIAM M. WARREN.

[We are more than glad to publish the foregoing in full in the hope that it may aid in calling attention to a subject that is of the utmost importance both to physicians and journalists—editors and publishers. The fact is that there are a number of these osteopathic fakirs practising medicine in this state—even in Buffalo—without license, and in open violation of the law. When our District Attorneys can nerve themselves to the attack we shall hope these and others, even of the regular profession, who are defying our practice law will be brought to bay and made to pay the penalty of the statute for their unprofessional actions. We warn all such, whether regulars or irregulars, that their time is coming—even near at hand. No man can legally practise medicine in this state unless he is *duly registered* and the law points out how this must be done.

To return to Mr. Warren's libel suit, we cannot doubt that he will make a successful defense against this latest and lowest form of pathy. Moreover, we hope the Michigan court that hears the case will draw the line sharply between regular medicine and fakes.—EDITOR.]

